# Chilean University Students’ Satisfaction With Online Learning During COVID-19 Pandemic: Demonstrating the Two-Layer Methodology

**DOI:** 10.3389/fpsyg.2022.887891

**Published:** 2022-07-27

**Authors:** Rodrigo Montero, René Gempp, Miguel Vargas

**Affiliations:** ^1^Facultad de Administración y Negocios, Universidad Autónoma de Chile, Santiago, Chile; ^2^Facultad de Administración y Economía, Universidad Diego Portales, Santiago, Chile; ^3^Facultad de Economía y Negocios, Universidad Andrés Bello, Santiago, Chile

**Keywords:** virtual classes, online learning, COVID-19, student satisfaction, satisfaction domains, two-layer model, Chile

## Abstract

Student satisfaction is a crucial determinant of success in online education, but studies on satisfaction with virtual classes during the COVID-19 outbreak are still scarce. This research contributes empirical evidence regarding the determinants of overall satisfaction with online classes and academic performance through the domain satisfaction approach. Additionally, we introduce to the psychological literature the two-layer model, a well-known econometric methodology, to estimate the effect of domain satisfaction while controlling the impact of unobserved individual differences. Our analyses are based on a cross-sectional sample of *n* = 648 Chilean university students (53.4% female) surveyed at the end of the first academic semester of 2021, during the lockdown mandated by the Chilean government due to the COVID-19 pandemic. Results show that satisfaction with the support provided by the university, satisfaction with learning, and satisfaction with the perceived quality of the online classes significantly explain the 68% of the variance of satisfaction with the virtual classes experience. Furthermore, satisfaction with academic performance is significantly explained by satisfaction with grades, learning, and the perceived quality of the online classes (R^2^ = .65). We also explore heterogeneous effects, separating them by gender and geographic area, and find that domains that systematically impact students’ satisfaction with online education are satisfaction with grades, learning, and the quality of classes.

## Introduction

The outbreak of COVID-19, declared a pandemic by the World Health Organization in March 2020 (WHO, 2020), had severe consequences for educational systems worldwide (Sarkar et al., 2021). Because of the higher transmissibility of the virus, many governments worldwide suspended in-person classes in schools and universities to curb the dissemination of the disease and implement online courses (Adedoyin and Soykan, 2020; Dhawan, 2020).

Chile was no exception, and, with varying degrees of success, universities had to adjust quickly to an online environment during the first quarter of 2020. Although some Chilean universities had experience in online learning, the country’s higher education system lacked prior experience in virtual classes. Therefore, moving to an online environment represented a considerable challenge, and the difficulties experienced impacted the teaching and learning process for students (Treviño et al., 2022).

By 2021 most Chilean universities had completed almost 2 years of online learning. There were some exceptions during the second semester of that year: some universities had a dual regime, mixing online learning with in-person classes (but with limited capacity per classroom, reaching few students), while other universities ruled that evaluations would be carried out in face-to-face mode (Valenzuela and Rodriguez, 2022).

Online learning represented an enormous challenge for the Chilean university system. On the one hand, many faculty members did not have sufficient mastery of digital tools for the correct transition to an online environment. Consequently, it was necessary to train instructors in digital environments and, at the same time, adapt the curriculum to cover the academic content in this new context (Treviño et al., 2022). On the other hand, many students lived in areas where internet connection quality was limited. This situation led most universities to decide that classes should be recorded and made available to students for viewing at any time (Valenzuela and Rodriguez, 2022).

For university students, the campus shutdown and the unplanned switch to remote learning impacted several aspects of the students’ experience in higher education, including satisfaction with online classes (She et al., 2021).

Student satisfaction, defined as students’ subjective evaluation of educational experience (Elliott and Shin, 2002; Weerasinghe and Fernando, 2017), is an outcome increasingly important in higher education because of its pivotal role in student success (Guo, 2016).

Indeed, pre-COVID evidence shows that student satisfaction is a crucial determinant of success in online education (Kuo et al., 2014; Alqurashi, 2019; Rabin et al., 2020). A few recent studies during the COVID-19 outbreak suggest the same conclusion (e.g., Gopal et al., 2021; Karadag et al., 2021; Sarkar et al., 2021), but the research in this regard is still scarce. In addition, most studies on student satisfaction conducted in psychology and education rely on multiple regression or Structural Equation Models (SEM) without properly controlling for endogeneity or omitted variables, a central concern in other fields of knowledge (Lütkepohl, 1982; Nakamura and Nakamura, 1998).

Accordingly, this paper aims to disentangle the factors that explain the satisfaction with online learning during the COVID-19 outbreak, among Chilean university students and introduce the two-layer model, an econometric methodology, as a robust tool for analyzing students’ satisfaction.

### Student Satisfaction With Online Learning During Campus Shutdown

Before the COVID-19 crisis, several studies addressed the determinants of satisfaction with online classes and compared them with in-person learning. For instance, McFarland and Hamilton (2005) explore the differences between students’ performance and satisfaction in traditional and online classes. Their results do not show significant differences in the degree of satisfaction with each kind of learning modality.

Other studies focused explicitly on online learning. Lee (2010) provides evidence for Korean and American students regarding online education support service quality, online learning acceptance, and satisfaction. One of its main results is that perceived online support service quality was a significant predictor of online learning acceptance and satisfaction for Korean and American students.

In the same vein, Lu and Chiou (2010), using data for Taiwan, proposed four predictors of e-learning satisfaction: interface friendliness (system quality with learner interface), content richness (information quality with content), perceived flexibility, and perceived community (service quality with personalization and learning community). All four factors had strong predictive power. Moreover, results suggested that a serious consideration of contingent variables (student job status and learning styles) is crucial for improving e-learning system satisfaction.

Furthermore, Schubert-Irastorza and Fabry (2011), investigates the factors that produce dissatisfaction in students of online academic programs. Study results suggested that negative student evaluations are most strongly influenced by lack of organization, lack of clarity, and insufficient feedback. Research by Kuo et al. (2013) and Kirmizi (2015) highlight that previous experience with technological tools and student readiness are relevant predictors of satisfaction with online education.

Other researchers addressed students’ satisfaction with Massive Open Online Courses (MOOCs). For example, Hew et al. (2020) reports that course instructor, content, assessment, and schedule are significant predictors of student satisfaction with the course.

Although the above studies and others conducted before the COVID-19 pandemic assist in the understanding of the factors influencing student satisfaction, their results cannot necessarily be applied in a generalized fashion to online classes during the campus shutdown due to the COVID-19 outbreak.

The fundamental difference between online classes pre- and post-COVID-19 is that before the pandemic students could choose online courses and programs, and universities were able to prepare them carefully. In other words, classes were likely better designed, instructors were better trained, and students were more intrinsically motivated to take online courses (Costa et al., 2021; Treviño et al., 2022; Valenzuela and Rodriguez, 2022). In contrast, the suspension of in-person classes due to the pandemic and the consequent switch to online learning was an unplanned, forced life event (Costa et al., 2021; Muthuprasad et al., 2021; Selvaraj et al., 2021).

For that reason, it is relevant to study the determinants of satisfaction with online courses triggered by COVID-induced lockdowns.

There are some recent studies aimed to address this issue. For instance, Gopal et al. (2021), using data from 574 Indian management students, found that the most important predictors of students’ satisfaction were the perceived quality of instructor, course design, instructor’s feedback, and student’s expectation.

Meanwhile, Karadag et al. (2021), using comprehensive data from nearly 15,000 students enrolled in 30 Turkish universities, report that universities with higher distance education capacities got higher satisfaction scores from their students.

In the Latin–American context, Jiménez-Bucarey et al. (2021) presents evidence regarding the satisfaction of Chilean medical students with the digital transformation process that was experienced due to the COVID-19 pandemic. Student satisfaction was measured across three dimensions: teacher quality, technical service quality and service quality. Using a structural equation model, the authors found that it is very important to improve the quality of technical service provided, in terms of teacher training, and the use of teaching strategies that promote student participation.

### A Two-Layer Approach to Student Satisfaction

A robust and helpful approach to assess the determinants of student satisfaction comes from the so-called aggregation approach, used in the happiness economics literature to investigate the determinants of subjective wellbeing (Rojas, 2006; Easterlin and Sawangfa, 2007; Kant et al., 2014; Loewe et al., 2014; Mahmud and Sawada, 2018; Montero and Miranda, 2020).

The rationale of the aggregation approach is to assume that the subjective wellbeing of the individual is the result of the satisfaction obtained in each of the domains that are relevant to their life (for example, work, family life, and health status, among others). Rojas (2006) uses an aggregation approach to analyze the determinants of life satisfaction in Mexico, where he showed that satisfaction in the personal, economic, health, work and family spheres were determinants of satisfaction with life. In a similar study using data for the United States, Easterlin and Sawangfa (2007) show that people’s satisfaction with their finances, their health, their work, and their family life were important to explain their life satisfaction.

Applying the same rationale to the satisfaction with online learning, the model to be estimated is:


(1) $$s_{i}=f\left(D_{1},D_{2},\ldots ,D_{k}\right)+u_{i}$$

where *s_i_* corresponds to overall satisfaction with online classes, *D_1_, D_2,_…, D_k_* correspond to satisfaction in each relevant domains for online classes experience, and *u_i_* is a well-behaved stochastic shock.

Note that the above formulation is equivalent to the well-known multiple regression or path analytic models in which global satisfaction is the dependent variable, satisfaction with domains of online experience are independent variables, and *u_i_* is typically known as an error term or unexplained variance. Indeed, the aggregation approach is the mechanism underlying the regression and SEM methods used in most studies on the determinants of satisfaction with online classes, reviewed in the preceding section.

An often-overlooked problem with the aggregation approach (and, therefore, with regression and SEM methods) is that non-modeled individual differences can bias parameter estimation. For example, people of different ages, gender, or ethnic groups might evaluate satisfaction with online classes differently. Even latent individual differences (e.g., personality traits) could also influence the results. For example, more agreeable people may assess the quality of service more benevolently, or more conscientious students may be more critical of the perceived quality of a class.

To overcome this problem, Van Praag et al. (2003) propose an improvement to the aggregation approach that they call the “two-layer model.” The first layer of the model establishes that overall satisfaction (for the purposes of this article this correspond to satisfaction with online learning) is the result of the satisfaction obtained in different domains, *plus* a variable *z* controlling for unobserved individual differences (e.g., personality traits). The second layer establishes that a set of exogenous variables determines each domain; then, from this layer, it is possible to create a proxy variable ($\hat{z}$) of the unobserved individual differences that predispose students to make specific satisfaction judgments.

Therefore, the benefit of this approach is that it allows the consideration of the unobservable variables that affect overall satisfaction, controlling the omitted variables bias that can arise when unobserved individual differences are not included. Notice that Van Praag et al. (2003), drawing from econometric literature, initially claim that *z* represents “*personality traits.*” However, we propose using the phrase “*unobserved individual differences*” instead because it better captures the underlying meaning of the proxy variable ($\hat{z}$) and is more consistent with the psychological and behavioral sciences literature.

More formally, the first layer of the model is given by:


(2) $$s_{i}=f\left(D_{1i},D_{2i},\ldots ,D_{ki},z_{i}\right)+u_{i}$$

where *z* is an unobservable variable that affects the subjective wellbeing.

For the second layer of the model, we assume that satisfaction in each domain depends on the objective situation of the individual (summarized by a vector of characteristics, *X*) and on their dispositional traits (*z*). Thus, traits are not observable and codetermine both *s* and *D_i_* (with *j = 1, 2, …, J*). Therefore, for each (J) domain satisfaction (*D_i_*):


(3) $$D_{ji}=h\left(X_{ji},z_{i}\right)+\epsilon_{i}$$

Remember that if equation (2) is estimated without controlling for unobserved individual differences (*z*), the estimates of the parameter for each domain satisfaction (D) will be biased and inconsistent.

Since, by definition, a measure of unobserved individual differences is not available in surveys, Van Praag et al. (2003) suggest constructing a proxy variable for *z*. That is the key ingredient of the two-layer method. The estimation of a proxy variable for z follows a three steps procedure.

First, we estimate equation (3) for each of the domains (“*J*” in total), obviously not including *z*, by ordinary least squares regression (OLS). In more familiar terms, we run an OLS regression for each domain, using domain satisfaction as the dependent variable and a set of characteristics, *X*, as predictors. Those Xs can be manifest individual differences, such as gender, age, socioeconomic status, etc.

Then, in the second step, we calculate the residuals of each estimated equation. Remember that regression residuals are simply the arithmetic difference between observed values and those predicted by the regression model. In this context, the OLS residuals represent estimates of the contribution of *z* to each domain.

Third, we extract the first principal component of the covariance matrix among those residuals. We use this first component as an instrumental variable representing the proportion of *z* common to all the domains.

This proxy variable ($\hat{z}$, since it is a proxy of *z*) is added as an additional regressor to equation (2), which allows us to assume that the error term (*u_i_*) will not be correlated with the variables that represent the domains (*D_k_*), and therefore, the parameter estimates will be consistent.

For the purposes of this research the variable *s* corresponds to the satisfaction of university students with online learning. On the other hand, the domains considered are the following: (1) satisfaction with the support provided by the university, (2) satisfaction with the dedication and interest of the professors, (3) satisfaction with the technological platform used by the university for virtual classes, (4) satisfaction with the quality of pedagogical material, (5) Satisfaction with the relationship with classmates, (6) satisfaction with the relationship with instructors, (7) satisfaction with grades obtained, (8) satisfaction with the level of learning achieved, and (9) satisfaction with the quality of online classes. Therefore, for this case *J* = 9.

## Materials and Methods

### Participants and Procedure

This study is part of a larger investigation aimed at understanding the explanatory factors of the college experience of Business and Accounting students. Consequently, we recruited undergraduate Business and Accounting students only *via* poster advertisements and mailing lists across three medium-size Chilean universities (Universidad Diego Portales, Universidad Autónoma de Chile, Universidad Nacional Andrés Bello) that offer these professional programs. The three universities have campuses in the capital of the country (Metropolitan area), and two of them also have campuses in other regions of Chile. An *a priori* power analysis with STATA 17 (StataCorp, 2021) revealed that a sample size of *n* = 635 participants was necessary to detect an R^2^ even as small as 2% with sufficient statistical power (1 − β = 0.80).

The students were invited to participate voluntarily and anonymously in an online survey on satisfaction with distance education during the COVID-19 lockdown. We did not offer them payment for participation.

The questionnaire was applied through the Qualtrics platform, at the end of the first academic semester of 2021 during the lockdown mandated by the Chilean government due to the COVID-19 pandemic.

As usual in this kind of study, the first screen of the online questionnaire presented a description of the research and an informed consent form, designed to confirm that the participant has been given all relevant information about the study and their role within it. They were also told that they could withdraw from the study at any time without consequence. Participants had to agree with the consent form before proceeding to the actual questions.

With the recruitment strategy described above, we completed a final sample of n = 648 participants after 1 month. The final sample includes 364 women (53.4%) and 302 men (46.6%) from the three universities (UDP = 42.75%; UA = 34.41%; UNAB = 22.84%), enrolled in business (74.23%) and accounting (25.77%). Most participants study and live in the Metropolitan area (79.48%), and they have an average age of 22.59 years (SD = 4.33).

The data that support the findings of this study are openly available in the Open Science Framework [OSF] at: https://osf.io/vytn8/?view_only=c6969b9501e24bec8ac9caf33127fa31.

### Measures

Each participant was asked to fill out an online questionnaire divided into two sections. The first section included questions regarding socio-demographic variables, required for estimating the first layer of method of Van Praag et al. (2003) method: university, undergraduate program (Business or Accounting), years in college (“*number of years that have passed since you entered college*”), geographical zone of residence (metropolitan area or other regions), age and gender.

Based on the studies mentioned early on satisfaction with online classes and drawing from desk-based reviews of student satisfaction surveys, the second section of our questionnaire comprised several questions to assess overall and domain satisfaction with remote classes.

To measure students’ overall satisfaction, we asked for academic satisfaction with online classes (“*what is your overall level of satisfaction with the virtual classes experience so far?*”) and current academic performance (“*What is your level of satisfaction with your current academic performance?*”). Both questions used a percentual response scale, ranging from 0 to 100, anchored from 0 = “*not satisfied at all*” to 100 = “*very satisfied.*”

For assessing satisfaction with specific domains of the virtual classes experience, we asked the respondents to rate their satisfaction with (1) the *support provided by the university*, (2) the *dedication and interest exhibited by the instructors*, (3) the *user-friendliness of technological platform used by the university for online classes*, (4) the *quality of pedagogical material (class notes, videos and slides)*, (5) the *relationship with classmates*, (6) the *relationship with instructors*, (7) the *grades obtained so far*, (8) the *level of learning achieved*, and with (9) the *quality of online classes sessions so far*. We also use a percentual scale for these questions, bounded from 0 = “*not satisfied at all*” to 100 = “*very satisfied.*”

On the type of questions used, we acknowledge that single-item scales do not have a good reputation among psychology researchers, and the discipline’s tradition is to prefer multi-item scales (Allen et al., 2022). Because in traditional measurement theory the items are supposed to represent a random selection from the hypothetical domain of indicators of the construct (Nunnally and Bernstein, 1994), multi-item scales could capture the whole complexity of any construct better than single-item scales. A related limitation is a lack of a measure of internal consistency reliability.

On the other hand, some authors consider the assertion that one *must* use multi-item measures as an urban legend (Boyd et al., 2005), and some others go even further, standing out that single-item scales have several advantages over multi-item measures, in terms of cognitive burden and readability (i.e., Matthews et al., 2022).

Our view is more aligned with a recent editorial in the *European Journal of Psychological Assessment*: although multi-item scales are undoubtedly superior, single-item scales are not automatically inferior to multi-item measures, and they are acceptable when constructs are unidimensional, clearly defined, and narrow in scope (Allen et al., 2022, p 3). Furthermore, multi-item scales are preferred, and evidence is needed to support using a single-item scale to measure a particular construct. In the specific case of the measurement of satisfaction with (any domain), past research support that single-item scales deliver comparable results to multi-item measures (e.g., Nagy, 2002; Dolbier et al., 2005; Cheung and Lucas, 2014; Mark et al., 2014; Montero and Rau, 2015, 2016; Jovanović, 2016; Sears et al., 2017; Fülöp et al., 2020; Jovanović and Lazić, 2020; Gempp and González-Carrasco, 2021). Several of these studies also report adequate test–retest coefficients for single-item measures.

To be clear, we are not claiming that single-item measures are better than or preferable to multi-item measures. We argue that, for measuring satisfaction, empirical evidence shows that single-item scales are equally valid and reliable as multi-item measures. Of course, this might not be the case in other research areas and for different types of constructs. The use of single-item scales is a matter that should always be based on the best available evidence.

Finally, since the discussion of the limitations and scope of single-item measures is beyond the scope of this paper, we recommend the editorial by Allen et al. (2022) and the references therein for a detailed review.

## Results

Table 1 presents the descriptive statistics of the sample in terms of subjective wellbeing. The first two columns show the average satisfaction with virtual classes and with academic performance. Then, the next nine columns show the average satisfaction with each of the nine domains already defined. Note that the scale for all the satisfaction measures ranges from 0 to 100.

**Table 1:** Descriptive statistics.

	*s*_1_	*s*_2_	*D*_1_	*D*_2_	*D*_3_	*D*_4_	*D*_5_	*D*_6_	*D*_7_	*D*_8_	*D*_9_
All (*n* = 648)	64.35 (27.97)	65.30 (26.96)	57.99 (28.99)	70.35 (24.23)	75.35 (23.81)	70.53 (24.32)	57.78 (31.08)	60.51 (29.58)	68.51 (23.85)	61.80 (27.65)	65.27 (25.94)
Men (*n* = 302)	62.51 (29.19)	63.66 (27.73)	58.63 (29.29)	69.69 (24.43)	75.55 (23.51)	69.09 (24.51)	56.49 (30.78)	57.57 (29.51)	68.30 (24.68)	60.67 (28.30)	62.90 (26.47)
Women (*n* = 346)	66.48 (26.53)	67.05 (26.16)	57.66 (28.79)	71.55 (23.70)	75.89 (23.31)	71.96 (23.88)	59.29 (31.06)	63.34 (29.34)	69.23 (22.59)	63.34 (26.70)	68.04 (24.78)
Metropolitan area = 1 (*n* = 515)	64.21 (27.63)	65.25 (26.82)	57.43 (29.19)	69.78 (24.19)	75.82 (24.12)	70.65 (24.20)	57.79 (31.02)	59.88 (29.40)	68.71 (23.55)	61.46 (27.42)	65.13 (25.95)
Metropolitan area = 0 (*n* = 133)	64.88 (29.32)	65.47 (27.60)	60.17 (28.24)	72.57 (24.33)	73.51 (22.56)	70.06 (24.86)	57.74 (31.44)	62.91 (30.23)	67.76 (25.02)	63.14 (28.59)	65.79 (26.02)

(s_1_) Satisfaction with virtual classes; (s_2_) Satisfaction with current academic performance; ($D_{1}$) satisfaction with the support provided by the university, ($D_{2}$) satisfaction with the dedication and interest of the professors, ($D_{3}$) satisfaction with the technological platform used by the university for online classes, ($D_{4}$) satisfaction with the quality of pedagogical material, ($D_{5}$) Satisfaction with the relationship with classmates, ($D_{6}$) Satisfaction with the relationship with instructors, ($D_{7}$) satisfaction with grades obtained, ($D_{8}$) satisfaction with the level of learning achieved, and ($D_{9}$) satisfaction with the quality of online classes. Estimates include dummy variables by university. All the measures range from 0 to 100. Standard deviations are in parenthesis.

In Table 1, it is possible to see that satisfaction with virtual classes is M = 64.35 points (SD = 27.97), which could be considered as a middle evaluation. Men make a lower evaluation (M = 62.51, SD = 29.19) than women (M = 66.48, SD = 26.53), and the difference is significant (*t* = −1.81, df = 646, *p* = 0.03, *d* = 0.14). On the other hand, there is no statistically significant difference between the mean level of satisfaction of students from Metropolitan and other regions (*t* = 0.25, df = 646, *p* = 0.60, *d* = 0.02).

The average satisfaction with academic performance is M = 65.3 points (SD = 26.96), that is, an assessment equivalent to the previous dimension. Again, males have a worst perception (M = 63.66, SD = 27.73) than females (M = 67.95, SD = 26.16), although the difference is barely significant (*t* = −1.59, df = 646, *p* = 0.05, *d* = 0.12). As in the case of satisfaction with virtual classes, there are no significant differences in satisfaction with academic performance between students from the Metropolitan and other regions (*t* = 0.08, df = 646, *p* = 0.53, *d* = 0.008).

Regarding the satisfaction with specific aspects of the online learning experience, the domain best evaluated is D3, the satisfaction with the technological platform used by the university for virtual classes (M = 75.35, SD = 23.81). On the other side, the worst evaluated aspect is D_5_, the satisfaction with the relationship with classmates (M = 57.78, SD = 31.08). This general pattern is also observed for men, although in the case of women, the less satisfactory domain is D_1_, the support provided by the university.

Regarding the Van Praag et al. (2003) two-layer method, Table 2 presents results of the first layer. Remember that the first layer involves estimating equation (2) by ordinary least squares (OLS) for each of the nine domains. The following controls have been incorporated for each of the domains: dummy for gender (1 = women; 0 = men), age, years in college (“*number of years that have passed since you entered college*”), dummy for undergraduate program (1 = business; 0 = accounting), dummy for geographical zone (1 = metropolitan area; 0 = other regions), and dummies by university (two dummy variables were included since the students in the sample come from three universities).

**Table 2:** Two-layer method, first layer: predictors of domain satisfaction.

Domain satisfaction									
Variable	$D_{1}$	$D_{2}$	$D_{3}$	$D_{4}$	$D_{5}$	$D_{6}$	$D_{7}$	$D_{8}$	$D_{9}$
Women = 1 (*n* = 346)	−1.789 (2.296)	1.059 (1.923)	1.197 (1.880)	2.309 (1.923)	2.358 (2.485)	4.414* (2.355)	0.544 (1.875)	1.939 (2.175)	4.697^**^ (2.027)
Age	0.670^**^ (0.302)	0.447^*^ (0.238)	0.396^*^ (0.222)	0.106 (0.246)	0.431 (0.319)	0.549^*^ (0.295)	0.647^***^ (0.199)	0.902^***^ (0.224)	0.444^*^ (0.255)
Years in college	−3.605^***^ (0.756)	−1.306^**^ (0.621)	−0.532 (0.591)	−2.139^***^ (0.642)	−1.083 (0.771)	−1.184 (0.740)	−0.817 (0.587)	−1.976^***^ (0.685)	−2.091^***^ (0.658)
Business program = 1 (*n* = 481)	−1.311 (2.748)	−4.386^*^ (2.335)	−3.148 (2.180)	−1.708 (2.288)	−4.800 (2.991)	−3.711 (2.877)	−1.594 (2.369)	−4.125 (2.713)	−4.584^*^ (2.474)
Metropolitan area = 1 (*n* = 515)	−2.847 (3.191)	−1.028 (2.722)	0.417 (2.572)	1.744 (2.730)	1.267 (3.511)	0.715 (3.307)	2.925 (2.677)	2.437 (3.135)	2.047 (2.910)
Constant	56.60^***^ (7.597)	68.10^***^ (6.152)	72.22^***^ (5.936)	72.46^***^ (6.510)	53.14^***^ (8.286)	51.42*** (7.600)	57.18^***^ (5.794)	51.82^***^ (6.417)	63.44^***^ (6.484)
N	648	648	648	648	648	648	648	648	648
R squared	0.040	0.020	0.023	0.026	0.010	0.029	0.019	0.040	0.034

* 10% significance level.

** 5% significance level.

*** 1% significance level.

The domains are as follows: ($D_{1}$) satisfaction with the support provided by the university, ($D_{2}$) satisfaction with the dedication and interest of the professors, ($D_{3}$) satisfaction with the technological platform used by the university for online classes, ($D_{4}$) satisfaction with the quality of pedagogical material, ($D_{5}$) Satisfaction with the relationship with classmates, ($D_{6}$) Satisfaction with the relationship with instructors, ($D_{7}$) satisfaction with grades obtained, ($D_{8}$) satisfaction with the level of learning achieved, and ($D_{9}$) satisfaction with the quality of online classes. Estimates include dummy variables by university. Standard errors are in parenthesis.

As previously explained, the OLS residuals are constructed from these estimates, and thus the common variance to all of them is extracted by principal components analysis. The first principal component constitutes a proxy for the variable *z,* which is then incorporated as an additional regressor when estimating equation (1).

Thus, Table 3 shows the results for the second layer, for two kinds of outcomes. For satisfaction with virtual classes and satisfaction with current academic performance, we presented the estimates by OLS of equation (1), controlling for the variable z as a regressor.

**Table 3:** Two-layer method, second layer: subjective wellbeing of students, controlling for z.

Variables	Satisfaction with virtual classes	Satisfaction with current academic performance
Satisfaction with the support provided by the university	0.0985^***^ (0.034)	−0.0179 (0.038)
Satisfaction with the dedication and interest of the instructors	−0.0468 (0.052)	−0.0846 (0.062)
Satisfaction with the technological platform used by the university for online classes	0.0833^*^ (0.048)	0.0351 (0.043)
Satisfaction with the quality of pedagogical material	−0.00727 (0.055)	−0.0371 (0.058)
Satisfaction with the relationship with classmates	0.0194 (0.038)	−0.0122 (0.038)
Satisfaction with the relationship with instructors	0.00911 (0.047)	0.0495 (0.052)
Satisfaction with grades obtained	−0.00223 (0.051)	0.514^***^ (0.051)
Satisfaction with the level of learning achieved	0.460^***^ (0.058)	0.308^***^ (0.064)
Satisfaction with the quality of online classes	0.393^***^ (0.060)	0.191^***^ (0.066)
z	−0.401 (1.563)	−0.174 (1.834)
Constant	0.559 (11.75)	3.182 (12.74)
*N*	648	648
R squared	0.677	0.648

* 10% significance level.

*** 1% significance level.

Standard errors are in parenthesis.

Regarding the satisfaction with virtual classes, it is worth highlighting the excellent adjustment that the model presents, evidenced by a 68% of explained variance. Considering the domains, satisfaction with the support provided by the university, satisfaction with learning, and satisfaction with the quality of online classes are the strongest determinants of overall satisfaction with virtual classes. Satisfaction with the technological platform used by the university also has a positive effect, but only at a significance level of 10%. Finally, it is possible to appreciate that the variable *z*, which controls the impact of unobserved individual differences, does not have a statistically significant effect.

Table 3 also shows the estimation of satisfaction with academic performance. Results reveal that the domains that positively and significantly (at 5%) affect satisfaction with academic performance are satisfaction with grades, satisfaction with learning, and satisfaction with the quality of online classes. Satisfaction with the support provided by the university is no longer relevant, and what matters is satisfaction with grades (which was not relevant to evaluate the experience with virtual classes).

Next, we explored the existence of heterogeneous effects, for which separate estimates were carried out by gender and geographical area. Results are presented in Tables 4, 5.

**Table 4:** Two-layer method: estimates separated by gender.

	Men (*n* = 302)		Women (*n* = 346)	
Variables	Satisfaction with virtual classes	Satisfaction with current academic performance	Satisfaction with virtual classes	Satisfaction with current academic performance
Satisfaction with the support provided by the university	0.0507 (0.047)	−0.0705 (0.053)	0.165^***^ (0.049)	0.0616 (0.053)
Satisfaction with the dedication and interest of the instructors	−0.0630 (0.073)	−0.0168 (0.080)	−0.0195 (0.076)	−0.149^*^ (0.086)
Satisfaction with the technological platform used by the university for online classes	0.132^*^ (0.079)	0.0674 (0.076)	0.0579 (0.063)	0.0168 (0.052)
Satisfaction with the quality of pedagogical material	−0.0492 (0.070)	−0.0366 (0.083)	0.0134 (0.089)	−0.0496 (0.080)
Satisfaction with the relationship with classmates	−0.0182 (0.056)	−0.0224 (0.054)	0.0636 (0.051)	0.0222 (0.055)
Satisfaction with the relationship with instructors	0.0241 (0.073)	0.0329 (0.071)	0.00250 (0.064)	0.0552 (0.074)
Satisfaction with grades obtained	0.0315 (0.076)	0.474^***^ (0.076)	−0.0344 (0.075)	0.555^***^ (0.072)
Satisfaction with the level of learning achieved	0.484^***^ (0.080)	0.300^***^ (0.087)	0.460^***^ (0.086)	0.337^***^ (0.092)
Satisfaction with the quality of online classes	0.258^***^ (0.090)	0.0941 (0.098)	0.498^***^ (0.083)	0.285^***^ (0.087)
z	1.960 (2.457)	1.814 (2.210)	−3.025 (2.349)	−2.612 (2.811)
Constant	9.492 (18.68)	9.493 (16.96)	−11.18 (17.50)	−7.159 (18.23)
N	302	302	346	346
R squared	0.738	0.705	0.631	0.610

* 10% significance level.

*** 1% significance level.

Standard errors are in parenthesis.

**Table 5:** Two-layer method: estimates separated by geographical area.

	Metropolitan area = 1 (*n* = 515)		Metropolitan area = 0 (*n* = 133)	
Variables	Satisfaction with virtual classes	Satisfaction with current academic performance	Satisfaction with virtual classes	Satisfaction with current academic performance
Satisfaction with the support provided by the university	0.101^**^ (0.039)	−0.00995 (0.045)	0.123^*^ (0.067)	−0.0176 (0.082)
Satisfaction with the dedication and interest of the instructors	−0.0130 (0.061)	−0.0624 (0.071)	−0.129 (0.121)	−0.159 (0.131)
Satisfaction with the technological platform used by the university for online classes	0.114^**^ (0.054)	0.0657 (0.048)	0.00623 (0.106)	−0.0341 (0.096)
Satisfaction with the quality of pedagogical material	−0.00934 (0.059)	−0.0484 (0.069)	0.0427 (0.157)	0.0692 (0.131)
Satisfaction with the relationship with classmates	0.00937 (0.042)	−0.00279 (0.044)	0.0868 (0.091)	−0.0525 (0.087)
Satisfaction with the relationship with instructors	0.00718 (0.053)	0.0436 (0.060)	0.0508 (0.095)	0.124 (0.111)
Satisfaction with grades obtained	0.0469 (0.058)	0.551^***^ (0.057)	−0.0978 (0.100)	0.443^***^ (0.123)
Satisfaction with the level of learning achieved	0.451^***^ (0.065)	0.314^***^ (0.073)	0.497^***^ (0.116)	0.315^**^ (0.145)
Satisfaction with the quality of online classes	0.409^***^ (0.063)	0.212^***^ (0.072)	0.394^**^ (0.163)	0.162 (0.173)
z	−1.250 (1.870)	−1.301 (2.351)	−0.625 (2.464)	0.984 (2.341)
Constant	−7.266 (14.27)	−4.880 (15.84)	4.611 (16.59)	10.08 (14.53)
N	515	515	133	133
R squared	0.684	0.641	0.673	0.687

* 10% significance level.

** 5% significance level.

*** 1% significance level.

Standard errors are in parenthesis.

In Table 4, results show that, for men, two domains influenced overall satisfaction with virtual classes: satisfaction with learning and satisfaction with the perceived quality of online classes. Satisfaction with the platform used by the university also has a positive impact, but significant only at 10%. In contrast, for women, satisfaction with the support provided by the university is a significant predictor.

Regarding satisfaction with current academic performance, Table 4 also shows that satisfaction with grades and with learning have a positive and statistically significant effect (at 5%) for men. The same occurs in the case of women, but satisfaction with the quality of the online classes is also added.

The estimates of the two-layer model, separated by geographical area, are presented in Table 5. For students in the Metropolitan area campuses, satisfaction with the support provided by the university, with the technological platform, with perceived learning and with the quality of online classes, are the most relevant determinants of overall satisfaction with virtual classes. On the other hand, for students from other regions, the determinants are the same except for the platform used by the university, which does not have a statistically significant impact.

For the satisfaction with current academic performance, the most relevant predictor among students from the Metropolitan area are grades, learning, and class quality. For students outside the Metropolitan area, the domains that explain satisfaction with current academic performance are grades and learning.

## Discussion

Table 6 summarizes results of the estimates. The symbol “**✓**” denotes that the effect is statistically significant.

**Table 6:** Satisfaction with online classes and with academic performance: summary.

	All		Men		Women		Metropolitan area = 1		Metropolitan area = 0	
Satisfaction with:	(a)	(b)	(a)	(b)	(a)	(b)	(a)	(b)	(a)	(b)
The support provided by the university	✓				✓		✓			
The dedication and interest of the instructors						✓				
The technological platform used by the university for online classes	✓		✓				✓			
The quality of pedagogical material										
Relationship with classmates										
Relationship with instructors										
Grades obtained		✓		✓		✓		✓		✓
The level of learning achieved	✓	✓	✓	✓	✓	✓	✓	✓	✓	✓
The quality of online classes	✓	✓	✓		✓	✓	✓	✓	✓	

(a) Dependent variable: satisfaction with virtual classes. (b) Dependent variable: satisfaction with current academic performance. (✓*) the domain is statistically significant in at least one of the models.*

First, we can highlight that only five of the nine domains have a positive and statistically significant effect, either on virtual classes satisfaction or on current academic performance.

Indeed, the domains that systematically have a positive impact on students’ satisfaction are satisfaction with grades (this domain is only relevant to explain satisfaction with current academic performance), satisfaction with learning, and satisfaction with the quality of online classes (these last two domains are relevant to explain both dimensions evaluated). On the other hand, there are two domains that only in certain cases present a statistically significant effect: satisfaction with the support provided by the university, and satisfaction with the technological platform used by the university.

A special mention is deserved by what happened with the (negative) effect that satisfaction with the dedication and interest of instructors has on satisfaction with the academic performance of female students. It is a result that can have multiple interpretations, but it certainly attracts attention. This result means that the more satisfied the female students are with the dedication and interest of the professors, the less satisfied they are with their current academic performance. We can hypothesize that instructors’ interest does not necessarily translate into higher performance, which damages the expectations of female students.

On the other hand, some domains are irrelevant when explaining satisfaction with overall online learning or current academic performance: satisfaction with the quality of the pedagogical material, satisfaction with the relationship with classmates, and satisfaction with the relationship they have with the instructors.

Another aspect systematically revealed by the estimates is that the variables associated with unobserved individual differences (variable *z*) do not have a significant effect in explaining the subjective wellbeing of students, with very specific exceptions.

Results should guide higher education institutions regarding which are the aspects that should be reinforced to improve the subjective evaluation that students make of online learning. Here it is possible to identify two variables. First, satisfaction with learning. Thus, the universities must guarantee that in the context of an online environment learning takes place on the part of the students. This is a challenge, because in this context the students interact less among themselves and with the instructor, and in fact, in general, they do not turn on their cameras.

The second variable is related with satisfaction with the quality of the online classes. To promote the quality of the classes, a permanent policy of support for instructors must be in place. Observing the instructor’s class will also be helpful in providing feedback. It is very necessary for instructors to be trained in the use of information and communication technologies since it will allow them to access various resources to improve the quality of their online classes.

Our findings should be viewed with caution. The estimates were carried out with a sample of students from three universities in Chile; therefore, the results of the study cannot be generalized to other samples. In this sense, it would be convenient to gather information from different countries, thus more fully understanding the determinants of satisfaction with the virtual academic experience of young people. We should also remember that we use single-item measures, whose limitations have already been discussed in section Measures.

It would be interesting for a possible future line of research to establish the effect that the academic performance of peers has on the subjective wellbeing associated with the online experience of young people. This is because it is widely documented that subjective wellbeing (satisfaction with life, for example) depends not only on one’s own monetary income but also on the monetary income of the group with which the individual is compared, the reference group (Ferrer-i-Carbonell, 2005; Card et al., 2012; Montero and Rau, 2015, 2016; Montero and Vásquez, 2015; Montero and Miranda, 2020). A similar phenomenon could occur in the context of learning, where the result of a peer affects one’s own subjective wellbeing.

## Conclusion

To conclude, we have investigated how online education has been perceived by undergraduate students in Chilean Universities. We have implemented an econometric method to control the effect that unobserved individual differences play in regard to students’ subjective evaluations. Results show, on one hand, that satisfaction with the support provided by the university, satisfaction with learning, and satisfaction with the quality of the online classes, are the most relevant domains to explain satisfaction with the virtual classes experience. On the other hand, satisfaction with academic performance is affected the most by domains such as satisfaction with grades, satisfaction with learning, and satisfaction with the quality of the online classes. In order to evaluate the existence of heterogeneous effects, estimates are provided, separating them by gender, and by geographic area. In this context, the domains that systematically have a positive impact on the subjective wellbeing of students are satisfaction with grades, satisfaction with learning, and satisfaction with the quality of classes.

Due to the relevance that online and hybrid education have attained, a trend that will continue growing even after the pandemic has come to an end, universities must understand how to deliver the best educational experience, particularly to those students belonging to the most vulnerable groups of society which present more difficulties accessing computers and Wi-Fi connections, something that would make the gap between the haves and the have-nots even wider. Our investigation has helped to cast light on some aspects related to this discussion and to provide insights to improve students’ online educational experience.

## Data Availability Statement

The data that support the findings of this study are openly available in the Open Science Framework [OSF] at: https://osf.io/vytn8/?view_only=c6969b9501e24bec8ac9caf33127fa31.

## Ethics Statement

Ethical review and approval was not required for the study on human participants in accordance with the local legislation and institutional requirements. The patients/participants provided their written informed consent to participate in this study.

## Author Contributions

All authors listed have made a substantial, direct, and intellectual contribution to the work and approved it for publication.

## Funding

MV received support from the Centro de Estudios de Conflicto y Cohesión Social, COES ANID/FONDAP/15130009. The support was to give money for data collecting and conference presentations.

## Conflict of Interest

The authors declare that the research was conducted in the absence of any commercial or financial relationships that could be construed as a potential conflict of interest.

## Publisher’s Note

All claims expressed in this article are solely those of the authors and do not necessarily represent those of their affiliated organizations, or those of the publisher, the editors and the reviewers. Any product that may be evaluated in this article, or claim that may be made by its manufacturer, is not guaranteed or endorsed by the publisher.
